# Special Issue “Mechanistic and Prognostic Biomarkers in Liver Diseases”

**DOI:** 10.3390/livers5040060

**Published:** 2025-11-24

**Authors:** Hartmut Jaeschke, Mitchell R. McGill

**Affiliations:** 1 Department of Pharmacology, Toxicology, and Therapeutics, University of Kansas Medical Center, Kansas City, KS 66160, USA; 2 Department of Environmental Health Sciences, Fay W. Boozman College of Public Health, University of Arkansas for Medical Sciences, Little Rock, AR 72205, USA; 3 Department of Pharmacology and Toxicology, College of Medicine, University of Arkansas for Medical Sciences, Little Rock, AR 72205, USA; 4 Department of Pathology, College of Medicine, University of Arkansas for Medical Sciences, Little Rock, AR 72205, USA

Basic science is critical for understanding fundamental biological processes and disease mechanisms. However, it is important to document that these mechanisms translate to humans and are relevant for human diseases. Although some mechanisms discovered in animal-based in vitro assays or in vivo models can be tested in human in vitro systems, such as primary human hepatocytes [1] or metabolically competent human liver cell lines [2,3], ultimately, evidence for these mechanisms needs to be obtained directly in humans. However, liver tissue from a living individual can only be obtained by biopsy or during surgery. This is a challenge because biopsy is contraindicated in some liver diseases, and even when it is medically justified, biopsy provides only a small amount of tissue, which may not be representative of the whole liver. Furthermore, the timing and context of biopsy or surgery are determined by patient care rather than research needs, so the conditions may not be ideal for procuring tissue for experimental investigations. In contrast, circulating biomarkers are easy to obtain and may be more likely to reflect dysfunction or injury of the entire organ. Mechanistic biomarkers provide insight into the mechanisms of liver diseases. For example, increases in plasma levels of bile acids indicate changes in metabolism, uptake, or excretion due to cell dysfunction or altered signaling, but bile acids themselves can also contribute to toxicity in cholestasis by damaging cell membranes [4,5]. Cytokines and chemokines may be generated in the liver and released by macrophages in response to many disease processes, but they can also further impact the mechanisms of liver disease by promoting inflammation [6,7]. Markers of necrosis are passively released due to loss of plasma membrane integrity and include the common clinical biomarkers alanine aminotransferase (ALT) and aspartate aminotransferase (AST) [8], but also high mobility group box 1 protein (HMGB1) [9], full-length keratin 18, and microRNA 122 (miR-122) [10–14]. On the other hand, apoptosis biomarkers include caspase-cleaved keratin 18 [10,13], active caspase enzymes themselves [15], and small DNA fragments [16]. Mitochondrial dysfunction or damage could be reflected by biomarkers such as the matrix enzyme glutamate dehydrogenase (GLDH) [13,15,17,18], mitochondrial DNA [13,15,17], acylcarnitines [19], and carbamoyl phosphate synthetase 1 (CPS-1) [20,21]. Plasma DNA fragments indicate nuclear damage during apoptosis or necrosis [22,23] but can also be an indirect biomarker of mitochondrial damage if the DNA fragmentation is caused by the release of mitochondrial proteins, such as endonuclease G and apoptosis-inducing factor, from damaged mitochondria, as in the specific case of acetaminophen overdose [15,23,24]. Besides mechanistic information, some of the mentioned biomarkers, including full-length keratin 18, GLDH, and miR-122, are also considered as diagnostic biomarkers allowing for an earlier and potentially more specific prediction of drug-induced liver injury [25]. After injury, the mechanisms of regeneration are critical for recovery and survival. Alpha-fetoprotein is considered a direct biomarker of regeneration and is clearly elevated after injury [8,26]. In addition, the recovery of plasma levels of proteins synthesized by hepatocytes, such as coagulation factors and complement proteins, that are depleted during the injury process, could be an indicator of regeneration [7,8]. Besides mediators synthesized in the liver or passively released by injured cells, the activation status of circulating immune cells could be a biomarker for the injury process or recovery. For example, the enhanced capacity of reactive oxygen formation and phagocytosis can demonstrate activation of neutrophils, and the timing of this effect during injury or regeneration can indicate a potential detrimental or beneficial effect of this inflammatory cell type on the injury or recovery process [27]. In addition, circulating biomarkers such as neopterin and soluble CD163 can indicate activation of macrophages [28]. These mechanistic biomarker studies in drug hepatotoxicity, in particular acetaminophen (APAP)-induced liver injury and acute liver failure (ALF), are strong examples and can be a guide for the expansion of these approaches to other acute and chronic liver disease processes, such as hypoxic hepatitis [13]. Similar or additional mechanistic or prognostic biomarkers may provide further insight into the pathophysiology of various human liver diseases, including Metabolic Dysfunction-Associated Steatotic Liver Disease (MASLD) and type 2 diabetes [29,30].

In contrast to mechanistic biomarkers, prognostic biomarkers predict the outcome of a liver disease process at early time points. The main purpose of these biomarkers is to guide clinical decision-making [8]. Again, studies in APAP-induced ALF can serve as an example. Once an injury has developed after an APAP overdose, the liver can either repair itself or progress to ALF and potentially death. In the latter case, a liver transplant may save the patient. However, due to the hyperacute nature of APAP-induced ALF, only a very narrow time window is available to decide whether to list the patient for transplantation. Thus, a readily measurable and accurate prognostic biomarker could greatly facilitate the selection of the appropriate patients for transplantation. Several of the above-mentioned mechanistic biomarkers have been evaluated as potential prognostic biomarkers [8]. Although it could be demonstrated in large groups of patients that higher mitochondrial dysfunction and damage correlate with negative outcomes [15,31], the substantial overlap of the datasets of survivors and non-survivors makes them less useful for individual cases. In contrast, recently described biomarkers such as CXCL14, angiopoietin-2, and lactate dehydrogenase (LDH) can predict who will need a liver transplant to survive with relatively high accuracy and close to the beginning or peak of liver injury [32–35]. The fact that these novel prognostic biomarkers can be readily measured by an ELISA or by enzyme kinetics within hours after blood sampling means that the results can be available in time to impact clinical decision-making. However, prognostic biomarkers for liver disease outcome can also in-volve other organs. For example, the plasma levels of kidney injury marker 1 (KIM-1) at the time of hospital admission are more than 500% higher in non-survivors than survivors after an APAP overdose, making KIM-1 an accurate prognostic biomarker for patients in need of a liver transplant to survive [36]. These are just a few examples of the clinical impact of prognostic biomarkers. Most acute and chronic liver diseases, including MASLD [37], alcoholic liver disease [38], fibrosis [39], liver cancer [40], and cholangiopathies [41,42], can benefit from the insight provided by prognostic biomarkers in terms of progression of the disease, monitoring effects of therapeutic interventions, and eventual clinical outcome.

Therefore, the objective of this Special Issue on “Mechanistic and Prognostic Biomarkers in Liver Diseases” (https://www.mdpi.com/journal/livers/special_issues/0757NW21HQ (accessed on 27 October 2025)) is to publish state-of-the-art reviews summarizing the newest developments in biomarker research by leading experts and attract additional reviews and original manuscripts that can further define the field, advance our understanding of the pathophysiology of human liver diseases, and identify novel therapeutic targets.

## Data Availability

No new data were created or analyzed in this study. Data sharing is not applicable to this article.
